# The crisis of maternal and child health in Afghanistan

**DOI:** 10.1186/s13031-023-00522-z

**Published:** 2023-06-12

**Authors:** Nancy Glass, Rabia Jalalzai, Paul Spiegel, Leonard Rubenstein

**Affiliations:** 1 Johns Hopkins School of Nursing, Baltimore, MD USA; 2 Johns Hopkins Bloomberg School of Public Health, Baltimore, MD USA; 3 Johns Hopkins Center for Humanitarian Health, Baltimore, MD USA; 4 Johns Hopkins University Hospital, Baltimore, MD USA; 5 Center for Human Rights and Public Health, Johns Hopkins Bloomberg School of Public Health, Baltimore, MD USA

**Keywords:** Maternal health, Humanitarian, Afghanistan, Women’s rights

## Abstract

**Background:**

The Taliban takeover in August 2021 brought global economic sanctions, economic collapse, and draconian restrictions on women’s freedom of movement, work, political participation, and education. This study examined Afghan health workers’ experiences and perceptions of availability and quality of maternal and child health care since then.

**Methods:**

We conducted a survey, using a convenience sample, of health workers from urban, semi-rural, and rural public and private clinics and hospitals across the 34 provinces, covering changes in working conditions, safety, health care access and quality, maternal and infant mortality as well as perceptions about the future of maternal and child health and health care. Interviews were conducted with a subsample of health workers to further explore their perceptions of changes in working conditions, quality of care, and health outcomes since the Taliban takeover.

**Results:**

131 Afghan practicing health care workers completed the survey. The majority were women (80%) working in facilities located in urban areas. Most female health workers (73.3%) reported that they have not always been safe when going to and from work; 81% because of harassment by the Taliban when they did not have male accompaniment. Almost half of the respondents (42.9%) reported a decrease in availability of maternal and child care and 43.8% stated that conditions for providing care were “worse” or “much worse” than before. Almost one-third (30.2%) indicated that changed working conditions negatively impacted their ability to provide quality care, and 26.2% reported an increase in obstetric and newborn complications. Health workers also reported (38.1% )an increase in sick child needs and an increase in child malnutrition (57.1%0. 57.1% reported decreases in work attendance and 78.6% a decrease in morale and motivation. Qualitative interviews (n = 10) of a subsample of survey participants expanded on these findings.

**Conclusion:**

The combination of economic collapse, lack of sustained donor support for health care and Taliban interference with human rights has severely compromised access and quality of maternal and child health care. Strong and concerted international pressure on the Taliban to respect women and children rights to essential health service is critical for the future of the Afghan population.

## Background

When the Taliban first seized power in 1996, Afghanistan’s healthcare system had already suffered from decades of war and limited investment by past governments [1]. The country had some of the highest rates of maternal, infant and child mortality in the world. During Taliban rule, morbidity, and mortality for women, infant and children worsened [2, 3]. The regime’s prohibition on women working outside the home or attending school, including female health care professionals, severely affected women’s lives, and undermined the health of the nation’s women and families [2, 3]. After the Taliban regime fell in 2001, the Ministry of Public Health (MOPH) inherited a healthcare system characterized by a shortage of human resources, particularly women, an infrastructure in ruins, and no sound data on the population’s health [1]. In response, the European Union (EU), United States Agency for International Development (USAID), and the World Bank provided sustained funding of hundreds of millions of US dollars for health services and partnered with the MOPH in creating an effective system of primary and secondary health care, training a new generation of healthcare professionals, including women [1, 4].

Initially, each donor provided the health service funding for an agreed set of provinces, but from 2013 to 2018, the System Enhancement for Health Action Transition (Sehatmandi) project supported a basic package of health services and essential package of hospital services under one umbrella through the Afghanistan Reconstruction Trust Fund platform. It was administered by the Ministry of Public Health, which contracted with non-governmental organizations (NGOs) to provide services.

As a result of significant investment in health, including recruitment and training of midwives, and Afghan leadership, despite an ongoing war that included violence inflicted on health personnel and facilities, Afghanistan reduced maternal mortality from approximately 1,450 deaths per 100,000 live births in 2000 to 638 deaths per 100,000 live births in 2017 [5, 6]; infant mortality from 88 deaths per 1,000 live births in 2001 to 45 deaths per 1000 live births in 2020 [5, 6] and under 5 years mortality reduced from 125 death per 1,000 in 2001 to 58 deaths per 1000 in 2020. [5, 6]. Access to health care improved dramatically for the whole society with more than 2,000 functioning healthcare facilities, including primary health centers, district hospitals, provincial hospitals, and specialized hospitals. Prior to regime change in August 2021, the vast majority (87%) of the Afghan population could reach a healthcare facility within two hours [7].

At the same time, in the roughly two decades before the Taliban takeover of Afghanistan in August 2021, respect for and protection of women’s rights became a focus of government and donor policy. It led to an increase in opportunities for women’s education and employment, including positions in government, the judiciary, academia, and health. The greatest impact was in urban areas, but also extended to places beyond them. Women were no longer required to be accompanied by a male escort when outside the home and were not subject to government-imposed dress codes.

Despite these improvements, the 2020 Human Development Index ranked Afghanistan 157th in gender equality, reflected in lack of reproductive health, women’s empowerment, and economic activity [8]. A 2018 survey of the problems facing women cited illiteracy, lack of educational opportunities and limits on women’s rights that reduce their opportunities to participate in governance and access justice [9]. The same year, more than a third of Afghan women give birth without skilled health attendant [10]. Rural women, especially, could not attend school, seek health care, or work without permission from a male. Women also had difficulty accessing health care due to the high costs in transportation and medicines [11]. In rural areas, poverty, conservative traditions, and inadequate health facilities impeded access [12].

### Health in the wake of regime change

After the Taliban took control of the country in August 2021, international donors initially declined to pay for any services channeled directly through the new regime, regardless of monitoring or accountability mechanisms established to ensure proper use of the funds. At the same time, the United States and many of its allies imposed stringent economic sanctions on the Taliban. They also adopted financial policies that hamstrung the functioning of Afghanistan’s central bank, thereby creating a liquidity crisis that brought the economy, based on cash transactions, to a halt. The crisis led to unemployment exceeding 70%. Food became unaffordable for many, and the threat of large-scale starvation loomed [13]. In an analysis conducted in early 2022, the United Nations (UN) Food and Agricultural Organization reported that 20 million people were going hungry, with certain pockets of catastrophic food insecurity, all fueled by the economic crisis combined with a lingering drought [14].

In an effort to ease the suffering of Afghans, first in late 2021, and again in February 2022, international governments provided humanitarian aid, including food aid, distributed through NGOs and UN agencies, and payments to NGOs operating health programs under the Sehatmandi program [15]. The World Bank released funds for education and health. In addition, individual money transfers from families and salary payments to some Afghan civil servants were approved [15]. The amounts provided, however, was a fraction of the large sum donors provided for health care and other services to Afghanistan before the Taliban takeover.

To prevent complete collapse of the health systems, donors and the UN and the International Committee of the Red Cross (ICRC) provided stop-gap funding for hospital services outside the Sehatmandi program. Donors supported operating expenses for 96 public hospitals and the ICRC supported an additional 33 hospitals. The World Health Organization (WHO) is supporting 17 hospitals and 189 primary health facilities [15].

Even so, the medical system has been suffering shortages of medications and supplies, infection control, staffing and supervision, even as new epidemics, including a large measles epidemic, broke out. In April 2022, more than a dozen UN human rights experts noted that the modifications and donor support for the Sehatmandi program taken to date had done little to restore cash-flow and relieve the humanitarian crisis, where 95% of the population having insufficient food, leading to serious increases in child malnutrition [13].

Further, despite the somewhat loosened rules and policies on money transfers, banks have been hampered in covering withdrawals because of lack of cash, even those based on electronic deposits and from humanitarian organizations [16]. Afghanistan remains cut-off from the international banking system, with dire consequences for the population. Negotiations among Western governments and the Taliban to provide a third-party substitute for the Central Bank of Afghanistan have taken place, with protections against Taliban interference, but no agreement had been reached [16].

The economic crisis exacerbated the suffering not only of people needing health care but their caregivers. In the months before the Taliban takeover, many health workers were not paid their salaries. When the liquidity crisis began and secure funding of health care ended, the at-best episodic nature of salary payments in the health sector continued, even after some of interim funding for health services arrived and the restrictions on cash transactions eased in early 2022. Many health workers left Afghanistan, exacerbating the stresses on health system functioning.

### The Taliban’s new attack on women’s rights

When the Taliban re-gained control of the country, it professed to respect the rights of women and girls and allow their education to continue. Initially, the Taliban gave contradictory signals about whether education for women and girls would be permitted, which led some schools to close while others remained open. On March 21, 2022, the Taliban committed to reopen all schools later that week. But within a few days thereafter, it disallowed all secondary education for women and girls. At the same time, it reinstated restrictions on women’s freedom of movement and enforced rules that women must be accompanied by a male escort, or *mahram* and are prohibited from entering parks, gyms and other public places. In December 2022, after the data for this study was collected, the Taliban closed universities to women and prohibited women from working in non-governmental and humanitarian organizations that are providing essential and lifesaving services to women and children throughout the country [17]. The Ministry of Public Health, Taliban spokesman said that the decree did not apply to the health sector, but the commitment is not in writing and its meaning remains unclear [18].

## Methods

The Johns Hopkins Center for Humanitarian Health, based at the Bloomberg School of Public Health, partnered with both a professional organization and NGOs to conduct a mixed method study. The study was conducted from November 2021 to April 2022 to examine the experiences and perceptions of health workers in public and private health systems in all of Afghanistan’s 34 provinces.

Data collection. The survey questions were developed in collaboration with Afghan health workers and colleagues who had worked in Afghanistan for several years. The team that developed the survey discussed the collection of demographic information from health workers such as age, education, province or health facility location. As this information has the potential for identifying participants, it was decided to not ask typical demographic questions. The survey questions focused on perceived changes in working conditions, safety, maternal and child health access and quality, and maternal and infant mortality. Afghan partners supported the recruitment of a convenience sample of health workers practicing in health clinics or hospitals in rural and urban communities throughout the 34 provinces and provided access to health care workers willing to complete the survey via Kobo Toolbox, an open access digital data collection platform. The survey was completed electronically in Pashto or Dari for those who felt that they had safe access. If participants did not feel their internet access was secure, they could complete the survey with a colleague via telephone. All participants provided informed consent by either responding YES to continuing participation on the Kobo platform or providing verbal consent to interviewer via telephone. Informed consent was obtained only after the participant received information on the study purpose, voluntary nature of participation, confidentiality and risk and benefits of participation. No personal or identifying information was include in the survey for security reasons. The survey was available through our partners for approximately one month.

Individual interviews were conducted with a subsample of the survey participants who volunteered to complete the interview via Zoom at a convenient time and in the language chosen by the participant. The interview guide focused on adding depth to the survey questions with specific focus on changes in working conditions, quality of care, and health outcomes since the Taliban takeover. Further, health workers discussed their concerns about education and mentorship for the next generation of health workers and the future of maternal and child health care in Afghanistan. The interviews continued until the team determined enough data has been collected to draw necessary conclusions, and any further data collection will not produce additional insight. As with surveys, no identifying or personal information was obtained during the interviews. Interviews were administered only after verbal informed consent was provided. The consent included audio recording for the interview thus allowing for transcription and then translation to English as needed for the analysis.

The surveys were analyzed using the software program SPSS 29.^1^

Two respondents were removed from the quantitative analysis because the participants reported not practicing prior to and since regime change. The qualitative interviews were analyzed with the assistance of a software program, MAXQDA2020.^2^ Two researchers read the transcribed transcripts and coded the transcripts based on themes concerning changes in working conditions, safety for health workers and patients, access to and quality of maternal and child health care, maternal and infant mortality and the future of health education and health care in Afghanistan since the regime change.

The Johns Hopkins Bloomberg School of Public Health’s Institutional Review Board approved the study, N0. 2001. 3

## Results

The surveys and interviews were conducted between February and April 2022. A total of 131 health workers participated in the surveys over the study period, 80% of the respondents were female. The majority of health professionals practiced in public health facilities in rural or semi-rural communities. A total of 10 health workers, all but one was female, completed the in-depth interviews.

### Deterioration of conditions for providing adequate care

The surveys and interviews revealed severe deterioration of conditions for providing care in the year after the Taliban takeover, and increased children and maternal deaths. Almost half (43.8%) of the health workers reported that the conditions for providing care were “worse” or “much worse” since August 2021. Over 40% (41.6%) reported that changes in working conditions or closure of the health facility had resulted in them not being able to practice.

Health workers described four reasons central to the severe decline in conditions: lack of regular salary payments, lack of medical supplies and medication, restrictions and harassment by the Taliban, and stresses associated with providing care with fewer staff.

*Lack of consistently paid salary.* Even after Western governments took steps to allow some financial transaction, almost all health workers said they had not been paid regularly. Although the vast majority (89.8%) of the health workers reported receiving some of their salary since 4 August 2021, the majority (57%) reported only receiving 50% or less of pay they were entitled to receive. In the interviews, health workers discussed how the economic crisis was having a significant impact on their families as they are now the only person in the household that can work. As one health worker stated:

“And there is no one except her (health worker), that she’s working to buy food, buy other things for the family, for the children, but you can imagine and, in that time, (August-November 2021), she cannot receive any salary. So, it’s really, really difficult.

“And many hospitals and care facilities, they have not even received their salaries, almost 4, 5 or 6 months. So that, puts an additional burden on families and on staffing primary care facilities”.

The restriction on money flows also had programmatic impacts. In the interview, health workers who had participated in donor funded health care system programs discussed job insecurity as the financial support from donors were month to month:

So, the projects [donor] funded will be approved monthly, the work plan of project. So, it’s really difficult for the person who is going to be working under this project. Every month, we are looking forward that our work plan is approved or not, do we receive our financial installment or not?

*Lack of supplies and medication.* Practicing health workers in rural, semi-rural and urban public and private health facilities consistently confirmed other reports of the devastating impact of loss of donor funds and the reduction in maternal and child health care and needed supplies and medications essential for quality of care to patients and families [12]. Almost half of the health workers (46.6%) in the survey reported a decrease in the availability of essential medicines to meet patient care needs.

One health worker described how a lack of medicines can lead to a loss in patient confidence and affects quality of care, stated:

I will counsel her for better nutrition behaviors to fulfill the deficiency of iron, but she will not be convinced, not listen to me, she will say that I need medicine because I’m in the situation that I need medicine and when the medicine is not available at the health facility, the next time they will not come to get the counseling. So, these are the things that really affects the quality of work.

Another health worker told me that a mother was bleeding and dying, but she didn’t have anything [no medicine]. She cannot do anything, she was just with the mother’s family, just sitting and seeing that the mother is dying, and we cannot do anything.

*Taliban restrictions and harassment.* Health workers experienced almost daily harassment and threat of violence by Taliban as they attempted to get to work and provide care to patients. The intimidation even took place during meetings with Taliban officials. One of the health workers described their first interaction with new Taliban leaders in the Ministry of Public Health:

They ask why do you come here? Where is your Mahram (male escort)? Where is your husband? You shouldn’t come without Mahram to my office (Ministry of Health). Very disrespectful with us (female health workers). So, all of this psychologically affects you a lot, that we are faced with such problems every day.

Almost three-quarters (73.3%) of health workers who participated on the survey reported that they have not always been safe when going to and from work. Of these, the majority (81%) said the lack of safety included being stopped and harassed by the Taliban because they did not have Mahram. In some cases, female health workers who participated in the interviews reported being beaten for failure to have a Mahram.

I want to add my experience because I was the project coordinator for one of our Health Centers. In the beginning of the new regime, two female health workers on their way to the Center were stopped by Taliban and they were asking where do you go and who are you? It was really very difficult for the women, and everyone was scared of them [Taliban]. So, they said we are going to the hospital, we are health care health workers but the two of them were beaten by Taliban because they did not have Mahram (male companion to accompany them to work), they did not have a Hijab. They (health workers) called me, I gave them off from work because they were not able to come to their center and provide services to the patient.

When stopped by police, female health care workers also described during the interview having to spend a significant amount of time trying to explain that they do not have a male escort in their home and that they are needed at the health facility. One health worker stated:

Now, the women are not allowed to get out of the house without Mahram. They [Taliban] are going to stop me on the street, I will show my ID card and that I’m working in hospital. Women who are jobless it is very difficult because when if they want to go to the clinic or the hospital if the Taliban stop and ask them for something, ID, they don’t have anything to show them. So people are very afraid of the Taliban. Women don’t want to be visible in the streets.

Another interview participant said:

I have more than 40 health care staff. They are coming to the hospital; they are faced with this Taliban people. They (Taliban) say we are not welcome at whatever job, or you cannot come to work without Mahram. We are explaining that we are health workers, we must go to the hospital. They become very serious in their discussion. They didn’t do any physical harm, but they shout at the women. We are praying.

Their situation has been especially precarious as more than half (51.4%) of female health workers reported on the survey to having to wait on the streets for long periods of time to get transportation to and from work. The long delay in getting transportation was described during the interview as taxi and bus drivers were fearful of picking up a woman without a male escort with Taliban patrolling the streets.

The interview participants detailed how harassment and lack of transportation could resulted in being late to relieve colleagues or depriving women and children of care. As one interview participant noted:

This is one of the really big problems especially for woman because when I’m coming to the office, I am late every day. I am late even if I get out of my house one or two hours earlier, there is no taxi or, and any other car, no driver is ready to pick up a woman without Mahram or without any other person. So, I, I have to wait for a long time on the streets on the roads for a car. I have to be ready to pay the cost of two or three people (the entire cab) and sit in back of the taxi, there is no woman allowed by Taliban in front of car.

*Workload stresses from lack of staff.* During interviews, health workers explained that many skilled health workers have left Afghanistan and many others who remains have ceased to work because of lack of pay or the harassment and violence they risk in going to work at health facilities. One health worker stated that in some facilities providing maternity care, “there is no trained midwife, no expert midwife.”

The decline in working conditions and regular pay has led many health workers to either quit or stop coming to work regularly. Among health workers, over half (57.1%) reported a decrease in work attendance and 78.6% described a decrease in morale and motivation. The vast majority (81%) of health workers in public health facilities reported that work attendance had decreased since August 2021. The findings were consistent for health workers working in urban, rural, and semi-rural facilities. Interview participants noted, “The stresses on those who came to work has increased.”

One health worker stated:

Health workers just write the prescription and nothing else, they don’t have enough time to take care or to give them [patient] advice on what they need.

They [health staff] start at 4pm, and they finish the shift at 8am. So it’s very long for one staff who is spending the night in the hospital for the patients. So in the morning, they will be really, really tired and they won’t have any more energy to spend. They try their best. But although they are trying their best, I mean, there will be some impact on the patient and on the process.

### Severe decline in availability and quality of care

Not surprisingly, the stresses of providing care without adequate staff, pay, supplies, and medication, while also subjected to Taliban harassment, led to declines in availability and quality of care at facilities. 40% (40.4%) of health workers reported that the availability of maternal and child health care has “decreased a little” to “decreased a lot” in their community since August 2021. A decline in availability of services has been noted by health workers across multiple geographic areas, Specifically, half (50%) of health workers in semi-rural settings, 43.9% of health workers practicing in rural settings, and one third (33.3%) of health workers practicing in urban settings reported a decline in availability of services. Almost one-quarter (24%) reported that because of the worsening conditions in health facilities they were not able to provide needed care for mothers and children. The quality of care has also suffered. About one-third (30.2%) of the health workers reported that the worsening of working conditions has negatively impacted their ability to provide quality care to women and children coming to the health facility.

Health workers described a decrease in availability of maternal, infant, and childcare, for example 42.9% of public and private health workers reported a decrease in availability of antenatal care. At the same time, these health workers reported a greater need, as one-third (38.1%) reported an increase in sick childcare needs, 57.1% reported an increase in malnutrition, and 26.2% reported an increase in obstetric and newborn complications. These impacts are in addition to declines in access to care are related to the economic crisis. The economic crisis has led mothers not to seek care for themselves or their families because they could not afford to travel or pay for medication. Additionally, women may delay health seeking because of risk of Taliban harassment if they came to the clinic unaccompanied by a male resulting in more complex and costly treatments.

As health workers described during the interviews:

Now the news is coming in because resources are scarce, and people prefer to take care of their patients at home. And when there are complications and there is definitely, that affects the survival.

When a health worker is only able to get to the health facility once a week, then of course, maybe she is missing many mothers, mothers with complications or something that we don’t even know.

Restrictions on movement have also affected women seeking health care. As one health worker noted:

Situation is really getting worse for woman day by day, day by day. There’re different restrictions for the woman, the Taliban announced that even if the woman wants to get out of the house her Mahram should be with them. So, it’s really difficult if she wants to move from the house and wants to go to the hospital or to the health center or to the office without Mahram it is really difficult.

### Perceived increase in maternal, infant and child mortality

Several health workers noted during the interviews that surveillance systems established to monitor maternal, infant and child mortality are not functioning, so the true impact on morbidity and mortality is not known. There are current efforts to restore sound morbidity and mortality surveillance in Afghanistan but in the meanwhile, the perceptions of health workers paint a disturbing picture.

More than one third (36.6%) of health workers reported that infant and child mortality has ‘increased a little’ to ‘increased a lot.’ As one health worker explained:

I have been in contact with health workers, there is malnutrition in children. Every day I’m visiting health facility or going to out to field. So, in 10 children, five or six children are malnourished and also the women, the women breastfeeding and pregnant woman.

Approximately one-third (31.4%) of health workers perceive that maternal mortality has increase since August 2021. One health worker stated:

I’m working in district hospital, but every week I’m the witness to at least two mothers that show up that are dying, we don’t have enough drugs.

Two months ago, one of the grandmothers told me that the health facility was near to her house, but because there was active fighting, her daughter lost two babies or twins babies. She was not able to go to health facility, so she delivered at home, she lost her two babies.

### Concerns for future of the Afghan Health Care System

Although stopgap measures have kept some of health facilities and most government hospitals functioning, health workers described ongoing challenges as limited access to quality care, lack of training opportunities for health workers, poor compensation, and limited freedom of movement for women. Further to these challenges, there is no global plan for financial support and the ongoing sanctions and economic policies are crippling the country’s economy. Health workers in this study concurred with other reports that without addressing these major impediments to health care women, infant and children will suffer, and many more lives will be lost [13].

Health workers also said that Afghanistan is facing a crisis in human resources for health because of lack of financial support for training and Taliban restrictions. The health workers emphasized the new regime’s restrictions, including prohibition of secondary and now university educations for girls and women, will have profound long-term impact on the health care and health outcomes for Afghan women, infant and children, as it will block the pipeline for female health workers, specifically midwife education and training.

As one health worker explained:

So, the health services will be of very poor quality, and we lack financial support and that will affect midwifery, or we can say medical pre-service education, that in future we will have a shortage of medical professionals especially midwives. As right now, we don’t have community midwifery programs, which is totally donor dependent program.

Another health worker added:

Similarly, in the government sector, the attrition or ‘brain drain’ is a big challenge in the entire country. Most of the experts and trained staff including nurses, midwives, and doctors have left the country.

## Discussion

This study found that a deterioration in human rights and donor support for health care threatens the safety of women and children in a country with one of the highest maternal mortality ratios in the world. Robust and comprehensive surveillance and population-based surveys are needed to determine whether, and to what extent, the perceptions of the health workers who participated in the research are borne out.

It is clear, though, that since its takeover in August 2021, the Taliban have oppressed girls and women by denying them education and employment, and routinely harassing and threatening violence against women. Inability to safely get to and from their jobs at health facilities to provide needed care to women and children, especially through Taliban restrictions in movement for women, have made the daily work of health care fraught with danger. These restrictions not only limit female health workers from providing care but influence women’s decisions to seek care for themselves and their children, potentially delaying access to care with negative outcomes including increased in maternal, infant and child mortality.

Practicing health workers expressed major concerns for the future of the health care systems and the ability to educate the next generation of health professions in Afghanistan. Health workers noted the long-term impact of financial policies on their ability to educate women as nurses, midwives and doctors with the skills and independence to function in diverse practice settings.

Since completing study data collection (February-April 2022), the Taliban have imposed even harsher regulations and restrictions that may impact working conditions in health facilities, quality of care and ability for patients and health professionals to get safely to and from the health facility. For instance, a recent Taliban regulation requires men and women patients be cared for in separate wards in rural provinces, this decree prohibits male physicians from providing care to women and vice versa, unless the care is an emergency [19–22]. There is little doubt these restrictions on care will further weaken maternal, infant and child health and the future health of the Afghan women and population. After the December decree prohibiting women from working in NGOs, health services were suspended delivery of health services at 288 health facilities, depriving 1.5 million of care [17]. Since then, the Taliban have stated that women could continue to work in health care, but the scope of the exception remains unclear [18].

A survey and focus groups conducted in four provinces after data collection for this study yielded resulted consistent with our findings. Specifically, extreme poverty, requirements of a Mahram, lack of availability of medications, and poor health quality remain severe obstacles for women seeking care. One consequence is that many women do not seek prenatal care and give birth at home instead of in a facility [18].

International donors, led by the United States together with the World Bank and other governments, deserve credit for providing significant humanitarian aid over the past year both as stopgap measures to avert mass starvation and to keep health services from collapsing.

The extraordinary commitments and resilience of health workers in Afghanistan, and the responsiveness of the ICRC, WHO and UNICEF, as well as some relaxation of sanctions rules to allow humanitarian aid, prevented many more deaths. Additionally, some health facilities may have planned for the potential of regime change and prepared by increasing their storage of needed supplies and medicines. For example, one health worker reported that their health facility had made plans and prepared for a potential change in the government as the security situation worsened in the months before the governments’ collapse by increasing their stock of needed supplies and essential medicines. The emergency measures also showed that fears that financial support of health care in Afghanistan could be accomplished without channeling funds through the Taliban-controlled agencies. Further, global economic policymakers, after strangling the economy of Afghanistan, including through preventing Afghanistan’s central bank from functioning, have sought to negotiate a solution that allow its critical function in a cash economy to function while not supporting the regime and ensuring accountability.

These actions, however, have not been sufficient to prevent severe suffering, including likely premature and unnecessary loss of life. As of January 2023, more than 28 million Afghans need urgent humanitarian assistance in order to survive and 17 million face acute hunger. Six million of whom experience emergency level of food insecurity (a step away from famine), the economic crisis including high unemployment has continued at a time of high inflation [22]. High maternal and child mortality, reduction in access to maternal and children health preventative and curative health services, and denial of women’s rights continue. Practicing health workers in rural, semi-rural and urban public and private health facilities consistently described the devastating impact of lack of pay, medical supplies and medications on availability and quality of care, confirming findings by others.

They also noted that deterioration of access to supplies and medication as well as less consistent salary payment began in the months prior to the government collapse. The worsening security situation in some provinces during that period resulted in the internal displacement of persons from provinces with ongoing conflict to provinces that were more peaceful that may have led to a weaker supply and could explain why the reports of lower access to medication and supplies after the Taliban takeover were not higher than the reported by health workers. What is needed now is a plan and funding or a sustainable health system, along with US and other donor financial support and economic policies, all with measures to keep funds away from the Taliban. Without addressing these major impediments to health care women, infant and children will suffer, and many more lives will likely be lost.

*Limitations.* The study has limitations. Specifically, when considering the findings, it is important to note that a sizable portion of the Afghan population does support the Taliban, especially in more rural areas of the country. For many, including health workers, who have lived and been impacted by conflict, trauma and loss, the Taliban brings an “end to the war,” so they are supportive of the regime. Further, there is a large and extensive intelligence network within the Taliban regime and even with the protections of an anonymous survey, there is likely a fear from some that may influence their responses on survey questions, such as not wanting to report information that could be considered negative towards the regime. Therefore, it is possible that some of the participants that answered questions about changes in maternal and child mortality may have chosen to select responses that are more positive than is the reality. In addition, we were not able to safely collect detailed information on the participants or the facilities and communities where they work, therefore the findings cannot be verified. Further, the qualitative and quantitative samples are limited and size and potential reach. For example, the qualitative interviews were conducted with health workers in primarily urban settings but did have ongoing contact with other health workers across the 34 provinces so could provide details during the interview about discussions they were throughout the country. The survey was completed by 131 health workers working in rural and urban areas across the country, thus the findings are not generalizable to the experiences of all health workers in rural or urban areas of Afghanistan. Although limitations exist, the findings are important as they present the reality on the ground from the providers experiences and perspective, perspectives essential to inform the needed changes in US financial policies for the Afghanistan population. Finally, the surveys and interviews took place in early 2022 and the situation may have changed for health workers. Given the new restrictions on women’s rights and the continuing lack of sustained funding for the health system, it appears that they still are germane today.

## Conclusion

The combination of donor support only for stopgap funding for Afghanistan’s health system and draconian restrictions by the Taliban on women’s rights are putting at great risk the modest gains in health and human rights for millions of women before its takeover of the government in August 2021. Both stained pressure for women’s rights and sustained funding for health care in Afghanistan can alleviate the crisis.

## Data Availability

and material: The safety and security of the survey and interview participants are the priority. We are not able to share the data with those outside the research team at the request of our Afghanistan colleagues. Investigators can contact nglass1@jhu.edu to request access to survey questions and interview guides.

## Footnotes

1 IBM Corp. Released 2021. IBM SPSS Statistics for Windows, Version 28.0. Armonk, NY: IBM Corp.

2 VERBI Software. (2020). MAXQDA 2020. Berlin, Germany: VERBI Software. Available from www.maxqda.com.

3 Johns Hopkins. Bloomberg School of Public Health IRB No. 2001, February 22, 2022, determination that the data collected was based on observations and expertise rather than personal experience, the study did not constitute human subjects research.

4 IBM Corp. Released 2021. IBM SPSS Statistics for Windows, Version 28.0. Armonk, NY: IBM Corp.
