# Correction to: The crisis of maternal and child health in Afghanistan

**DOI:** 10.1186/s13031-023-00529-6

**Published:** 2023-06-22

**Authors:** Nancy Glass, Rabia Jalalzai, Paul Spiegel, Leonard Rubenstein

**Affiliations:** 1 Johns Hopkins School of Nursing, Baltimore, MD USA; 2 Johns Hopkins Bloomberg School of Public Health, Baltimore, MD USA; 3 Johns Hopkins Center for Humanitarian Health, Baltimore, MD USA; 4 Johns Hopkins University Hospital, Baltimore, MD USA; 5 Center for Human Rights and Public Health, Johns Hopkins Bloomberg School of Public Health, Baltimore, MD USA

**Correction to:** ***Confl Health*** **17, 28 (2023)**

10.1186/s13031-023-00522-z

The original publication of this article contained an incorrect author name. The incorrect and correct information is listed in this correction article, the original article has been updated.

- The incorrect author name is: Rabia Jalazai.
- The correct author name is: Rabia Jalalzai.

